# Urinary tartaric acid as a biomarker of wine consumption and cardiovascular risk: the PREDIMED trial

**DOI:** 10.1093/eurheartj/ehae804

**Published:** 2024-12-18

**Authors:** Inés Domínguez-López, Rosa M Lamuela-Raventós, Cristina Razquin, Camila Arancibia-Riveros, Polina Galkina, Jordi Salas-Salvadó, Ángel M Alonso-Gómez, Montserrat Fitó, Miquel Fiol, José Lapetra, Enrique Gómez-Gracia, José V Sorlí, Miguel Ruiz-Canela, Olga Castañer, Liming Liang, Lluis Serra-Majem, Frank B Hu, Emilio Ros, Miguel Ángel Martínez-González, Ramon Estruch

**Affiliations:** Polyphenol Research Group, Departament de Nutrició, Ciències de l’Alimentació i Gastronomía, Facultat de Farmacia, Universitat de Barcelona (UB), Av. de Joan XXII, 27-31, Barcelona 08028, Spain; Institut de Nutrició i Seguretat Alimentària (INSA), Universitat de Barcelona (UB), Santa Coloma de Gramanet 08921, Spain; CIBER Fisiopatología de la Obesidad y Nutrición (CIBEROBN), Instituto de Salud Carlos III, Monforte de Lemos 3-5, Pabellón 11, Madrid 28029, Spain; Polyphenol Research Group, Departament de Nutrició, Ciències de l’Alimentació i Gastronomía, Facultat de Farmacia, Universitat de Barcelona (UB), Av. de Joan XXII, 27-31, Barcelona 08028, Spain; Institut de Nutrició i Seguretat Alimentària (INSA), Universitat de Barcelona (UB), Santa Coloma de Gramanet 08921, Spain; CIBER Fisiopatología de la Obesidad y Nutrición (CIBEROBN), Instituto de Salud Carlos III, Monforte de Lemos 3-5, Pabellón 11, Madrid 28029, Spain; CIBER Fisiopatología de la Obesidad y Nutrición (CIBEROBN), Instituto de Salud Carlos III, Monforte de Lemos 3-5, Pabellón 11, Madrid 28029, Spain; Department of Preventive Medicine and Public Health, University of Navarra, IdiSNA, Irunlarrea 1, Pamplona 31008, Spain; Polyphenol Research Group, Departament de Nutrició, Ciències de l’Alimentació i Gastronomía, Facultat de Farmacia, Universitat de Barcelona (UB), Av. de Joan XXII, 27-31, Barcelona 08028, Spain; Institut de Nutrició i Seguretat Alimentària (INSA), Universitat de Barcelona (UB), Santa Coloma de Gramanet 08921, Spain; Polyphenol Research Group, Departament de Nutrició, Ciències de l’Alimentació i Gastronomía, Facultat de Farmacia, Universitat de Barcelona (UB), Av. de Joan XXII, 27-31, Barcelona 08028, Spain; Institut de Nutrició i Seguretat Alimentària (INSA), Universitat de Barcelona (UB), Santa Coloma de Gramanet 08921, Spain; CIBER Fisiopatología de la Obesidad y Nutrición (CIBEROBN), Instituto de Salud Carlos III, Monforte de Lemos 3-5, Pabellón 11, Madrid 28029, Spain; CIBER Fisiopatología de la Obesidad y Nutrición (CIBEROBN), Instituto de Salud Carlos III, Monforte de Lemos 3-5, Pabellón 11, Madrid 28029, Spain; Human Nutrition Unit, Biochemistry and Biotechnology Department, Pere Virgili Medical Research Institute (IISPV), Sant Joan University Hospital, University Rovira i Virgili, Reus, Spain; CIBER Fisiopatología de la Obesidad y Nutrición (CIBEROBN), Instituto de Salud Carlos III, Monforte de Lemos 3-5, Pabellón 11, Madrid 28029, Spain; Bioaraba Health Research Institute, Osakidetza Basque Health Service, Araba University Hospital, University of the Basque Country UPV/EHU, Vitoria-Gasteiz, Spain; CIBER Fisiopatología de la Obesidad y Nutrición (CIBEROBN), Instituto de Salud Carlos III, Monforte de Lemos 3-5, Pabellón 11, Madrid 28029, Spain; Unit of Cardiovascular Risk and Nutrition, Hospital del Mar Medical Research Institute (IMIM), Barcelona, Spain; CIBER Fisiopatología de la Obesidad y Nutrición (CIBEROBN), Instituto de Salud Carlos III, Monforte de Lemos 3-5, Pabellón 11, Madrid 28029, Spain; Institute of Health Sciences, University of the Balearic Islands and Hospital Son Espases, Palma de Mallorca, Spain; CIBER Fisiopatología de la Obesidad y Nutrición (CIBEROBN), Instituto de Salud Carlos III, Monforte de Lemos 3-5, Pabellón 11, Madrid 28029, Spain; Department of Family Medicine, Research Unit, Sevilla Primary Health Care District, Sevilla, Spain; Department of Preventive Medicine, University of Malaga, Malaga, Spain; Department of Preventive Medicine, University of Valencia, Spain; CIBER Fisiopatología de la Obesidad y Nutrición (CIBEROBN), Instituto de Salud Carlos III, Monforte de Lemos 3-5, Pabellón 11, Madrid 28029, Spain; Department of Preventive Medicine and Public Health, University of Navarra, IdiSNA, Irunlarrea 1, Pamplona 31008, Spain; CIBER Fisiopatología de la Obesidad y Nutrición (CIBEROBN), Instituto de Salud Carlos III, Monforte de Lemos 3-5, Pabellón 11, Madrid 28029, Spain; Unit of Cardiovascular Risk and Nutrition, Hospital del Mar Medical Research Institute (IMIM), Barcelona, Spain; Department of Biostatistics, Harvard T.H. Chan School of Public Health, Boston, MA, USA; Department of Epidemiology, Harvard T.H. Chan School of Public Health, Boston, MA, USA; Institute for Biomedical Research, University of Las Palmas de Gran Canaria, Las Palmas, Spain; Department of Epidemiology, Harvard T.H. Chan School of Public Health, Boston, MA, USA; Department of Nutrition, Harvard T.H. Chan School of Public Health, Boston, MA, USA; Channing Division of Network Medicine, Department of Medicine, Harvard Medical School and Brigham and Women’s Hospital, Boston, MA, USA; CIBER Fisiopatología de la Obesidad y Nutrición (CIBEROBN), Instituto de Salud Carlos III, Monforte de Lemos 3-5, Pabellón 11, Madrid 28029, Spain; Institut d’Investigacions Biomèdiques August Pi i Sunyer (IDIBAPS), Barcelona 08036, Spain; CIBER Fisiopatología de la Obesidad y Nutrición (CIBEROBN), Instituto de Salud Carlos III, Monforte de Lemos 3-5, Pabellón 11, Madrid 28029, Spain; Department of Preventive Medicine and Public Health, University of Navarra, IdiSNA, Irunlarrea 1, Pamplona 31008, Spain; CIBER Fisiopatología de la Obesidad y Nutrición (CIBEROBN), Instituto de Salud Carlos III, Monforte de Lemos 3-5, Pabellón 11, Madrid 28029, Spain; Department of Internal Medicine, Hospital Clinic, IDIBAPS, University of Barcelona, Rosselló 149-153, Barcelona 08036, Spain

**Keywords:** Wine, Tartaric acid, Biomarker, Cardiovascular disease, Mediterranean diet

## Abstract

**Background and Aims:**

Moderate wine consumption has been associated with lower cardiovascular disease (CVD) risk in older populations. However, wine consumption information through self-reports is prone to measurement errors inherent to subjective assessments. The aim of this study was to evaluate the association between urinary tartaric acid, an objective biomarker of wine consumption, and the rate of a composite clinical CVD event.

**Methods:**

A case-cohort nested study was designed within the PREDIMED trial with 1232 participants: 685 incident cases of CVD and a random subcohort of 625 participants (including 78 overlapping cases). Wine consumption was registered using validated food frequency questionnaires. Liquid chromatography-tandem mass spectrometry was used to measure urinary tartaric acid at baseline and after one year of intervention. Weighted Cox regression models were used to estimate hazard ratios (HRs) of CVD.

**Results:**

Tartaric acid was correlated with self-reported wine consumption at baseline [r = 0.46 (95% CI 0.41; 0.50)]. Five categories of *post hoc* urinary tartaric acid excretion were used for better representation of risk patterns. Concentrations of 3–12 and 12–35 μg/mL, which reflect ∼3–12 and 12–35 glasses/month of wine, were associated with lower CVD risk [HR 0.62 (95% CI 0.38; 1.00), *P* = .050 and HR 0.50 (95% CI 0.27; 0.95), *P* = .035, respectively]. Less significant associations between self-reported wine consumption and CVD risk were observed.

**Conclusions:**

Light-to-moderate wine consumption, measured through an objective biomarker (tartaric acid), was prospectively associated with lower CVD rate in a Mediterranean population at high cardiovascular risk.

## Structured Graphical Abstract

**Structured Graphical Abstract:**
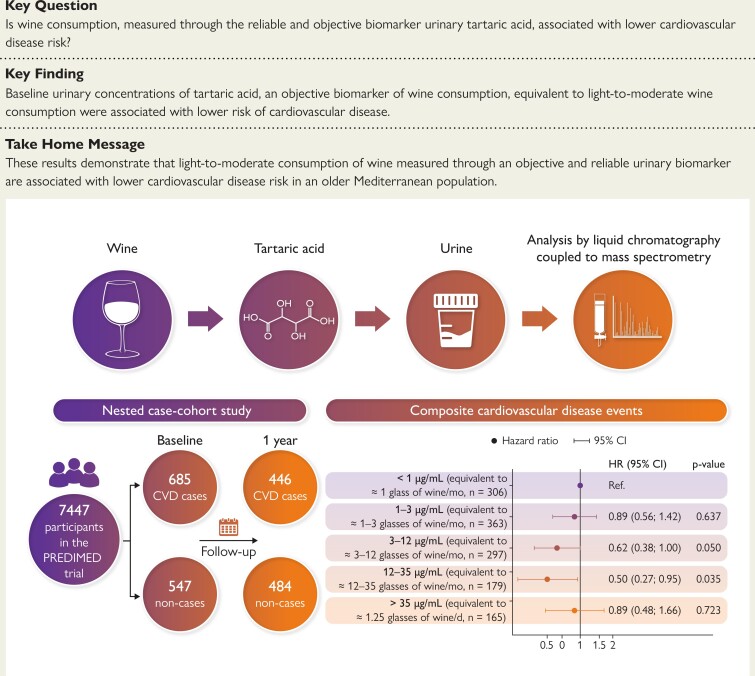
Overview of the experimental determination of tartaric acid and design of the nested case-cohort study. Main findings of the relationship between tartaric acid and CVD risk. CI, confidence interval; CVD, cardiovascular disease; HR, hazard ratio.

**See the editorial comment for this article ‘Wine consumption and cardiovascular health: the unresolved French paradox and the promise of objective biomarkers’, by G. de Gaetano *et al*., https://doi.org/10.1093/eurheartj/ehae726.**

## Introduction

Cardiovascular disease (CVD) stands as the leading global cause of mortality, with an estimated 17.9 million deaths each year, making up 32% of total global fatalities.^1^ There is a great interest in finding effective prevention strategies to reduce this burden and, accordingly, nutritional interventions have received increasing attention. Numerous epidemiological studies have suggested a relationship between higher adherence to the Mediterranean diet (MedDiet) and lower risk of developing CVDs.^2,3^ The landmark PREDIMED study is the largest clinical trial conducted to evaluate the effects of the MedDiet.^4^

Within the framework of the MedDiet, moderate consumption of alcohol, particularly wine, is thought to be one of the factors contributing to the cardioprotective effects of this dietary pattern.^5^ However, even though the health effects of wine have been studied for decades, there still is an ongoing debate regarding its potential advantages when consumed in moderate or low amounts.^6^ It is important to highlight that epidemiologic studies assessing the role of wine on CVD rate usually rely on self-reported information on wine consumption, potentially leading to measurement errors and impairing a correct quantification of intake due to potential misreporting also in relation with the perceived desirability of alcohol intake.^7^ Therefore, using an objective biological marker may enhance the accuracy of evaluating wine consumption. Tartaric acid is primarily produced in grapes and is synthesized very rarely by other plant species.^8^ Thus, tartaric acid emerges as a valuable short-term biomarker (several days to a week) for assessing wine consumption, provided that the intake of grapes and their derivatives is excluded. Indeed, prior studies from our laboratory have confirmed its usefulness as a reliable and objective biomarker of wine consumption.^9,10^

Using a case-cohort study nested within the PREDIMED trial, we analysed baseline levels and one-year changes in urinary tartaric acid and examined whether low-to-moderate levels of wine consumption as estimated by this biomarker were associated with a reduced rate of CVD events.

## Methods

### Study design

A prospective case-cohort analysis was conducted utilizing baseline and one-year data from the PREDIMED (PREvención con DIeta MEDiterránea) study. This large, parallel-group, multicentre, randomized, controlled intervention trial with a mean follow-up of 4.8 years assessed the impact of the MedDiet enriched with extra-virgin olive oil or nuts on the incidence of CVD.^4^ The characteristics of the trial were described in Supplementary data online, *Material S1*.

The case-cohort design included all incident cases of CVD with available urine samples, and a random subsample of 10% of the PREDIMED trial participants (referred to as the subcohort). Therefore, a total of 1232 participants were assessed, 685 incident cases of CVD and a random subcohort of 625 participants, including 78 overlapping incident CVD cases. A flowchart with this information is shown in Supplementary data online, *Figure S1*. Cases corresponded to participants who developed a clinical cardiovascular event (cardiovascular death, myocardial infarction, stroke, or heart failure) during the active trial or an extended follow-up period. Follow-up was based upon regular visits and review of medical records. Only 7.0% of participants were lost to follow-up for 2 or more years by the end of the trial (1 December 2010). During a median follow-up of 9.0 years of the extended follow-up period, 222 cases of heart failure, 138 cases of non-fatal myocardial infarction, 190 cases of non-fatal stroke, and 286 cardiovascular deaths were documented.

### Ascertainment of cardiovascular disease cases

In each recruitment centre, medical professionals who were blinded to the intervention conducted annual reviews of all participants’ medical records to investigate potential CVD events. To ascertain incident cases, four sources of information, also blinded to the intervention, were utilized: continuous communication with participants, interactions with their family physicians, annual examination of medical records, and consultation of the National Death Index. Subsequently, anonymized data were forwarded to a blinded central Event Ascertainment Committee, which conducted the final event adjudication.

### Urinary tartaric acid

Biological samples were collected after an overnight fast, coded, and stored at −80°C until analysis. Tartaric acid in urine was determined following a validated stable-isotope dilution liquid chromatography-electrospray ionization-tandem mass spectrometry (LC-ESI-MS/MS) method with minor modifications.^11^ Further details can be found in Supplementary data online, *Material S2*.

### Statistical analyses

Participants were divided into five categories according to their raw levels of urinary tartaric acid at baseline (<1 µg/mL, 1–3 µg/mL, 3–12 µg/mL, 12–28 µg/mL, and >28 µg/mL). These five categories were *post hoc* selected based on previous knowledge on meaningful thresholds to provide clearer insights into wine consumption.^12^ Baseline characteristics of participants are presented as means + standard deviation (SD) for continuous variables and percentages for categorical variables. We used one-factor analysis of variance (ANOVA) to assess baseline differences in continuous variables across categories of tartaric acid, and χ^2^ tests for categorical values.

Individual baseline values of tartaric acid were normalized and scaled in multiples of 1 SD with Blom inverse normal transformation due to high biological variability.^13^ Changes in tartaric acid (one-year value minus the baseline value) were calculated, and the resulting difference was also normalized and scaled. The transformed tartaric acid values were used when tartaric acid was analysed as a continuous variable (μg/mL per 1 SD).

Multivariable linear regressions were used to assess the relationship between self-reported wine consumption and urinary tartaric acid. Self-reported wine consumption, obtained through the validated food frequency questionnaire (FFQ), was analysed both as a continuous variable (per 1 SD increment) and using tertiles. Tartaric acid was introduced as a continuous variable (z-scaled). Three models of increasing complexity were designed. Model 1 was adjusted for age and sex. Model 2 was further adjusted for smoking (three categories), educational level (five categories), physical activity, body mass index (BMI), hypertension, dyslipidaemia, and diabetes. Model 3 was additionally adjusted for total energy intake, MedDiet adherence (not considering wine), and consumption of grapes and raisins (explaining 0.6% of total variance of urinary tartaric acid). Robust variance estimators were used in all models to account for potential clustering effects by recruitment centre. We estimated the correlation between urinary tartaric acid (1 SD increment in its transformed concentration) and self-reported wine consumption (1 SD increment) using Pearson’s correlation coefficients with their 95% confidence intervals (CIs).

The analysis for the receiver operating characteristic (ROC) curve was obtained through logistic regression using a dichotomous variable (consumers/non-consumers of wine) as the dependent variable and transformed concentrations of tartaric acid at baseline as the predictor. The logistic regression model was presented without adjustment and adjusted for the aforementioned variables.

We used Cox regression models with Barlow weights (to account for oversampling cases) to calculate the hazard ratios (HRs) and their 95% CI for the risk of the composite of CVD outcome in the extended follow-up period associated with successive categories of urinary tartaric acid concentration. The composite of CVD outcomes includes heart failure, acute myocardial infarction, stroke, and cardiovascular death. Baseline concentrations of urinary tartaric acid were analysed using the five categories with the cut-off points previously described (<1 µg/mL, 1–3 µg/mL, 3–12 µg/mL, 12–28 µg/mL, and >28 µg/mL), selecting as reference the group with <1 µg/mL. The equivalence of tartaric acid to glasses of wine was estimated based on excretion levels observed in a previous clinical trial conducted by our research group.^9^ Three adjustment models of increasing complexity in the Cox model were designed based on *a priori* identified confounders. Model 1 was also adjusted for age and sex. Model 2 was additionally adjusted for smoking, educational level, marital status, physical activity, BMI, waist-to-height ratio, hypertension, dyslipidaemia, diabetes, family history of CVD, and recruitment centre. Model 3 was further adjusted for energy intake, MedDiet adherence (excluding wine), and consumption of grapes and raisins. Models were stratified by centre, sex, and quartiles of waist-to-height ratio using the Stata command *strata*. In addition, we conducted the same analyses with self-reported wine consumption, adjusting for the same variables. The cut-off points for self-reported wine consumption were established at <1 glass/month, 1–3 glasses/month, 3–12 glasses/month, 12–35 glasses/month, and >35 glasses/month. The same categories and adjustment models were used to assess all-cause mortality as the outcome, after excluding the overlapping subjects in the subcohort.

We conducted stratified analyses by sex, baseline type 2 diabetes, and intervention group (both MedDiet interventions and low-fat control group) as *a priori* defined and assessed potential interactions with these variables using the likelihood ratio test. We also used restricted cubic splines with four knots to explore the shape of the dose–response relationship between baseline urinary tartaric acid and incident CVD. To test for non-linearity, we used the likelihood ratio test, comparing the model with only the linear term and the model with the linear and the cubic spline terms. These spline models were adjusted for the same potential confounders as the main Cox regression analyses at baseline.

*P*-values of <.050 were considered statistically significant. All statistical analyses were performed with Stata 16.0 (Stata Corp LP, College Station, TX, USA).

## Results

### Baseline characteristics

*Table 1* shows the baseline general characteristics of the 1232 participants (657 women and 575 men with a mean age of 68 years) according to baseline categories of urinary tartaric acid excretion. Median (Q1–Q3) baseline concentrations of tartaric acid in urine in each of these five categories were 0.7 (0.5–0.8), 1.6 (1.2–2.2), 5.4 (3.9–8.3), 21.4 (16.0–27.2), and 75.6 (49.2–117.3) µg/mL. As expected, wine consumption increased with higher levels of urinary tartaric acid. Categories with higher excretion of tartaric acid at baseline included more men and a higher proportion of current smokers. Participants with higher urinary tartaric acid also engaged in more physical activity, and their total energy intake was higher. As expected, participants with higher tartaric acid excretion also reported higher consumption of grapes and raisins, despite the extremely low intake of these fruits. Intake of other beverages that might contain tartaric acid, such as grape juice, was negligible. The dietary intake of participants at baseline according to categories of baseline tartaric acid excretion is detailed in Supplementary data online, *Table S1*. The consumption of dairy products was lower in categories with higher urinary tartaric acid excretion, while meat intake was higher among these participants. Individuals with tartaric acid levels ranging from 12 to 35 µg/mL exhibited a slightly higher intake of virgin olive oil.

**Table 1:** General characteristics of the study population at baseline (*n* = 1232)

	<1 µg/mL (*n* = 291)	1–3 µg/mL (*n* = 340)	3–12 µg/mL (*n* = 281)	12–35 µg/mL (*n* = 164)	>35 µg/mL (*n* = 156)	*P*-value
Urinary tartaric acid, µg/mL^a^	0.7 (0.5–0.8)	1.6 (1.2–2.2)	5.4 (3.9–8.3)	21.4 (16.0–27.2)	75.6 (49.2–117.3)	
Age, years	68.9 + 6.1	68.0 + 6.2	68.4 + 6.1	69.2 + 6.0	67.5 + 6.3	.054
Women, *n* (%)	187 (64.3)	204 (60.0)	150 (53.4)	61 (37.2)	55 (35.3)	<.001
BMI, kg/m^2^	29.9 + 4.2	30.3 + 3.7	29.9 + 3.6	29.9 + 3.4	29.9 + 3.4	.653
Diabetes mellitus, *n* (%)	175 (60.1)	190 (55.9)	142 (50.5)	84 (51.2)	73 (46.8)	.074
Dyslipidaemia, *n* (%)	199 (68.4)	236 (69.4)	194 (69.0)	118 (72.0)	98 (62.8)	.205
Hypertension, *n* (%)	240 (82.5)	290 (85.3)	235 (83.6)	132 (80.5)	131 (84.0)	.373
Cancer, *n* (%)	8 (2.8)	12 (3.5)	10 (3.6)	3 (1.8)	5 (3.2)	.789
Neurodegenerative disease, *n* (%)	1 (0.3)	0 (0)	2 (0.7)	1 (0.6)	1 (0.6)	.589
Educational level, *n* (%)						.511
Low	234 (80.4)	267 (78.5)	221 (78.7)	123 (75.0)	116 (74.4)	
High and medium	57 (19.6)	73 (21.5)	60 (21.4)	41 (25.0)	40 (25.6)	
Smoking habit, *n* (%)						<.001
Current smokers	33 (11.3)	36 (10.6)	33 (11.7)	23 (14.0)	45 (28.9)	
Former smokers	77 (26.5)	77 (22.7)	77 (27.4)	62 (37.8)	52 (33.3)	
Total energy intake, kcal/day	2205 + 567	2283 + 634	2316 + 629	2346 + 601	2410 + 647	.011
Physical activity, METS·min/day	214 + 231	215 + 209	270 + 255	256 + 237	265 + 257	.006
Family history of early-onset CHD, *n* (%)	65 (22.3)	78 (22.9)	67 (23.8)	30 (18.3)	29 (18.6)	.681
Intervention group, *n* (%)						.026
MedDiet + EVOO	92 (31.6)	123 (36.2)	93 (33.1)	70 (42.7)	51 (32.7)	
MedDiet + nuts	93 (32.0)	103 (30.3)	94 (33.5)	47 (28.7)	54 (34.6)	
Control diet	106 (36.4)	114 (33.5)	94 (33.5)	47 (28.7)	51 (32.7)	
Self-reported wine consumption, mL/day	15 + 37	28 + 52	63 + 96	114 + 127	151 + 159	<.001
Grapes and raisins consumption (g/d)	12 + 22	12 + 22	15 + 31	15 + 28	20 + 34	.020

Values are percentages for categorical variables and means ± SD for continuous variables. One-ANOVA factor was used for continuous variables, and a χ^2^ test was used for categorical variables. *P* < .05 was considered significant. The displayed *n* does not include overlapping subjects in the subcohort.

BMI, body mass index; METS, metabolic task equivalents; CVD, cardiovascular disease; MedDiet, Mediterranean diet; EVOO, extra-virgin olive oil.

^a^Tartaric acid concentrations are presented with the interquartile range (Q1–Q3).

### Tartaric acid as biomarker of wine consumption

The associations between wine consumption and baseline tartaric acid excreted in urine are presented in Supplementary data online, *Table S2*. Tartaric acid was positively associated with the consumption of wine when they were both analysed as continuous variables in the fully adjusted model [*β* = 0.47 (0.41; 0.53) µg/mL per 1 SD, *P* < .001]. When the relationship was assessed using tertiles of wine consumption instead of the SD, similar, highly significant, results were obtained. Participants in the highest tertile of wine consumption had higher urinary tartaric acid levels [*β* = 0.95 (0.78; 1.12) µg/mL per 1 SD, *P* < .001]. Supplementary data online, *Figure S2* presents the multivariable regression between baseline wine consumption and urinary tartaric acid excretion adjusted for potential confounders. The Pearson correlation coefficient between urinary tartaric acid and wine consumption was 0.46 (0.41; 0.50). Supplementary data online, *Figure S3* shows the ROC curve analysis, where it can be observed that urinary tartaric acid predicted wine consumption with an area under the curve of 0.70 (0.67; 0.73) changing to AUC = 0.79 (0.77; 0.82) after adding the other covariates.

### Baseline urinary tartaric acid and cardiovascular disease

Associations between baseline urinary tartaric acid and the rate of subsequent CVD events are presented in *Table 2*. Compared with participants who excreted <1 µg/mL of tartaric acid, those who excreted 3–12 µg/mL of tartaric acid in urine (approximately reflecting light consumption of wine) exhibited a significantly lower risk of CVD after adjusting for potential confounders [HR 0.62 (95% CI 0.38; 1.00), *P* = .050]. Participants who excreted 12–35 µg/mL of tartaric acid (approximately reflecting a moderate consumption of wine—up to 1 drink/day) also presented a significantly lower risk of CVD as compared to the reference group [HR 0.50 (95% CI 0.27; 0.95), *P* = .035]. No significant associations were observed for categories of higher or lower excretion of tartaric acid. When analyses were stratified by sex (*P*_for interaction_ = .52), the inverse association with light and moderate consumption of wine remained significant only in men [HR 0.41 (95% CI 0.20; 0.84), *P* = .015 for 3–12 µg/mL, and HR 0.31 (95% CI 0.12; 0.79), *P* = .014 for 12–35 µg/mL], whereas only a non-significant tendency towards lower rates was found in women with light consumption. Notwithstanding, these differences between men and women may be due to the lower number of CVD events in women. In any case, it should be acknowledged that no interaction was found between categories of baseline tartaric acid excretion and sex. To ensure that tartaric acid excretion was not associated with an increased risk of other causes of death, we conducted a similar survival analysis with long-term all-cause mortality (up to 31 December 2020, with a mean follow-up of 12.6 years) as the outcome. As it is shown in Supplementary data online, *Table S3*, our analysis with all-cause mortality did not reveal any significant harmful association for ranges of tartaric acid between 3 and 35 µg/mL (approximately reflecting low-to-moderate wine intake).

**Table 2:** Risk of cardiovascular disease by categories of baseline urinary tartaric acid concentrations for the total population and stratifying by sex

	Tartaric acid (µg/mL) per 1 SD		<1 µg/mL (*n* = 291)	1–3 µg/mL (*n* = 340)		3–12 µg/mL (*n* = 281)		12–35 µg/mL (*n* = 164)		>35 µg/mL (*n* = 156)	
	HR (95% CI)	*P*-value		HR (95% CI)	*P*-value	HR (95% CI)	*P*-value	HR (95% CI)	*P*-value	HR (95% CI)	*P*-value
Total											
Cases	685		169	188		143		94		91	
Person-years	6412		1403	1752		1612		886		759	
Model 1	0.94 (0.83; 1.07)	.371	1 (Ref.)	0.93 (0.66; 1.30)	.657	0.68 (0.48; 0.97)	.033	0.68 (0.46; 1.02)	.064	1.03 (0.68; 1.57)	.878
Model 2	0.91 (0.76; 1.09)	.294	Ref.	0.88 (0.56; 1.40)	.599	0.62 (0.39; 0.99)	.047	0.53 (0.29; 0.97)	.038	0.91 (0.49; 1.68)	.752
Model 3	0.91 (0.75; 1.09)	.301	Ref.	0.89 (0.56; 1.42)	.637	0.62 (0.38; 1.00)	.050	0.50 (0.27; 0.95)	.035	0.89 (0.48; 1.66)	.723
Men											
Cases	353		69	83		75		64		62	
Person-years	2641		428	627		654		483		449	
Model 3	0.82 (0.63; 1.06)	.136	Ref.	0.60 (0.27; 1.34)	.213	0.41 (0.20; 0.84)	.015	0.31 (0.12; 0.79)	.014	0.54 (0.23; 1.28)	.162
Women											
Cases	332		100	105		68		30		29	
Person-years	3771		975	1125		958		403		311	
Model 3	0.92 (0.69; 1.22)	.546	Ref.	0.97 (0.52; 1.80)	.912	0.52 (0.25; 1.08)	.080	0.69 (0.29; 1.67)	.413	1.03 (0.37; 2.92)	.951

The *n* does not include overlapping subjects in the subcohort. Model 1 was adjusted for age and stratified by sex. Model 2 was further adjusted for smoking, marital status, physical activity, educational level, BMI, waist-to-height ratio, hypertension, dyslipidaemia, diabetes, and family history of CVD, and stratified by sex, quartiles of the waist-to-height ratio, and recruitment centre. Model 3 was additionally adjusted for total energy intake, MedDiet adherence (not considering wine), and consumption of grapes and raisins. The *P-*value for interaction (sex ∗ category of the joint combination of baseline levels of tartaric acid, with 4 degrees of freedom) was derived from a Cox model adjusted as model 3, and it was not statistically significant *P*_for interaction_ = .52.

HR, hazard ratio.

**Figure 1:**
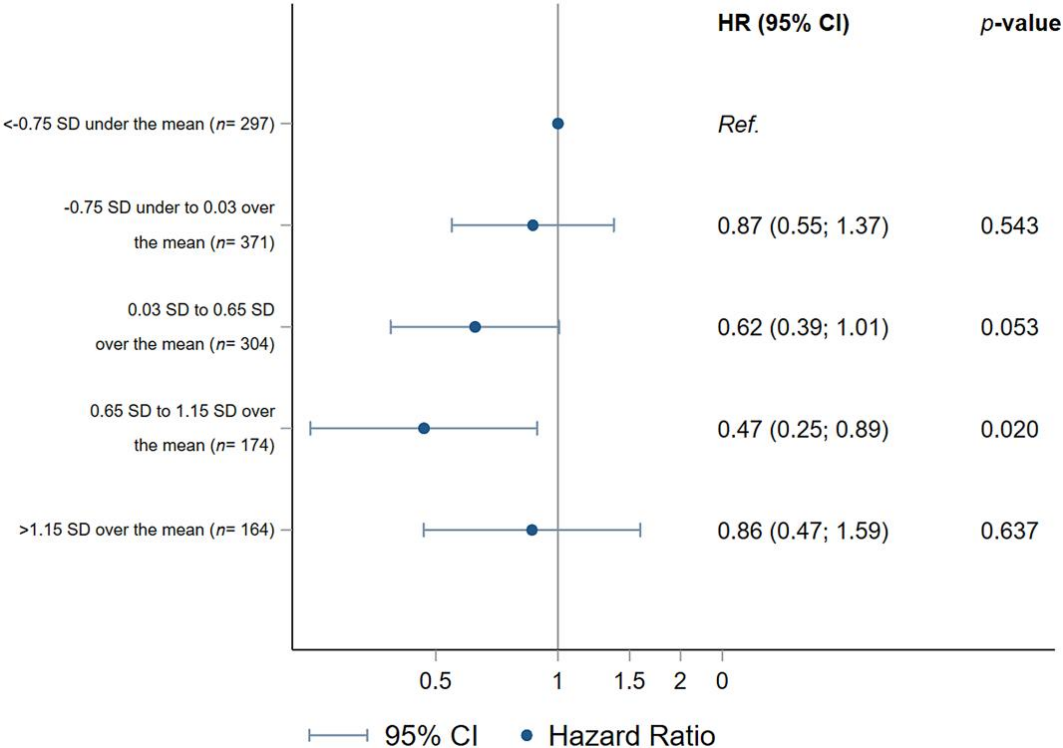
Multivariable adjusted HRs (95% CI) of CVD by categories of baseline urinary tartaric acid. CI, confidence interval; HR, hazard ratio; CVD, cardiovascular disease

**Figure 2:**
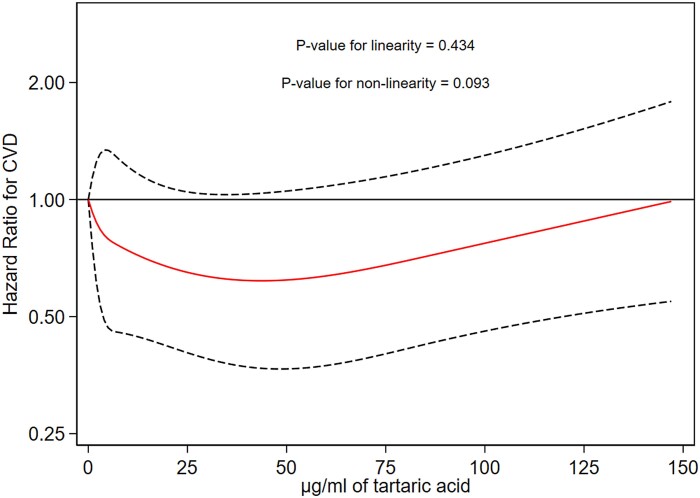
Multivariate-adjusted relation of baseline tartaric acid with CVD risk. Associations were evaluated with the use of restricted cubic splines. The solid lines represent the central risk estimate and the dotted lines represent the 95% confidence intervals (CIs)

The relationship between baseline urinary tartaric acid (per SD) and CVD for all participants is depicted in *Figure 1*. We observed that participants in the range of 0.65–1.15 SD above the mean (equal to 12.5–35.4 µg/mL of tartaric acid in urine) presented a significantly lower rate of CVD as compared to those with < −0.75 SD under the mean, whereas the same tendency was observed for the range of 0.03–0.65 SD over the mean (equal to 3.0–12.4 µg/mL of tartaric acid). In *Figure 2*, we used models with restricted cubic splines adjusted for the same potential confounding factor mentioned above to account for non-linear associations. However, no significant non-linear associations were found.

Supplementary data online, *Table S4* illustrates the association between self-reported wine consumption, assessed through the FFQ, and CVD. In contrast to the analysis involving urinary tartaric acid, categories reflecting lower wine consumption included a larger number of participants, potentially indicative of underreporting in self-assessment. Consequently, as anticipated, none of the categories demonstrated a reduced rate of developing CVD when compared to the reference group. The category of occasional consumption of wine (1–3 glasses/month) presented the highest CVD rates, significantly higher than the reference category (<1 glass/month) and also higher than the upper category of wine consumption (>35 glasses/month).

We also assessed the risk of CVD incidence by categories of baseline urinary tartaric acid according to diabetes status in Supplementary data online, *Table S5* (*P*_for interaction_ = .056). In participants with diabetes, tartaric acid concentrations equivalent to light consumption of wine (3–12 µg/mL) were associated with lower rate of CVD as compared to levels < 1 µg/mL [HR 0.45 (95% CI 0.21; 0.95), *P* = .036]. On the other hand, no association was found in participants without diabetes at baseline.

### Changes in urinary tartaric acid and cardiovascular disease

Some supplementary materials (see Supplementary data online, *Tables S6–S11* and Supplementary data online, *Figure S4*) show the characteristics of the population according to 1-year changes in tartaric acid, as well as their relationship with the rate of CVD, as ancillary analyses.

### Urinary tartaric acid and individual components of the composite cardiovascular disease endpoint

*Table 3* shows the risk for each individual component of the composite CVD endpoint (heart failure, myocardial infarction, stroke, or CVD death), according to baseline urinary tartaric acid concentrations. We observed a significant inverse association between baseline tartaric acid (per 1 SD) and the rate of myocardial infarction [HR 0.70 (95% CI 0.50; 0.97), *P* = .031]. In the analysis by categories of tartaric acid excretion, participants in the group of >35 µg/mL of urinary tartaric acid presented lower myocardial infarction rates as compared to the reference group [HR 0.26 (95% CI 0.07; 0.97), *P* = .045]. However, no differences were observed for the rates of any other individual component.

**Table 3:** Risk of cardiovascular events by categories of baseline urinary tartaric acid concentrations

	Tartaric acid (µg/mL) per 1 SD		<1 µg/mL (equivalent to ≈1 glass of wine/month, *n* = 291)	1–3 µg/mL (*n* = 340)		3–12 µg/mL (*n* = 281)		12–35 µg/mL (*n* = 164)		>35 µg/mL (*n* = 156)	
	HR (95% CI)	*P*-value		HR (95% CI)	*P*-value	HR (95% CI)	*P*-value	HR (95% CI)	*P*-value	HR (95% CI)	*P*-value
Heart failure											
Cases	198		47	60		42		22		27	
Model 1	0.95 (0.79; 1.13)	.542	1 (Ref.)	1.11 (0.69; 1.76)	.671	0.73 (0.44; 1.21)	.223	0.62 (0.34; 1.12)	.111	1.21 (0.68; 2.18)	.517
Model 2	0.88 (0.65; 1.19)	.421	Ref.	1.16 (0.56; 2.41)	.689	0.76 (0.35; 1.65)	.136	0.38 (0.13; 1.06)	.065	0.93 (0.34; 2.56)	.888
Model 3	0.90 (0.66; 1.23)	.517	Ref.	1.13 (0.53; 2.39)	.748	0.76 (0.34; 1.68)	.492	0.39 (0.13; 1.14)	.084	0.99 (0.36; 2.72)	.981
MI											
Cases	116		30	28		30		16		12	
Model 1	0.86 (0.70; 1.06)	.164	1 (Ref.)	0.71 (0.40; 1.28)	.258	0.77 (0.43; 1.37)	.371	0.62 (0.30; 1.26)	.187	0.58 (0.27; 1.25)	.164
Model 2	0.69 (0.50; 0.96)	.025	Ref.	0.58 (0.26; 1.32)	.192	0.65 (0.29; 1.47)	.297	0.32 (0.11; 0.99)	.048	0.25 (0.07; 0.93)	.038
Model 3	0.70 (0.50; 0.97)	.031	Ref.	0.62 (0.27; 1.43)	.265	0.67 (0.29; 1.56)	.353	0.30 (0.09; 1.08)	.066	0.26 (0.07; 0.97)	.045
Stroke											
Cases	150		40	41		26		23		20	
Model 1	0.90 (0.74; 1.10)	.316	1 (Ref.)	0.84 (0.51; 1.38)	.485	0.54 (0.31; 0.94)	.028	0.75 (0.41; 1.37)	.355	0.90 (0.48; 1.70)	.755
Model 2	0.91 (0.65; 1.27)	.579	Ref.	0.61 (0.28; 1.34)	.218	0.54 (0.26; 1.12)	.099	0.83 (0.31; 2.20)	.708	1.01 (0.35; 2.89)	.988
Model 3	0.91 (0.65; 1.27)	.569	Ref.	0.63 (0.28; 1.42)	.265	0.54 (0.25; 1.15)	.109	0.81 (0.28; 2.34)	.701	0.94 (0.33; 2.68)	.905
Death from CVD											
Cases	238		56	63		47		36		36	
Model 1	1.01 (0.84; 1.22)	.897	1 (Ref.)	0.97 (0.60; 1.57)	.907	0.66 (0.40; 1.10)	.109	0.74 (0.42; 1.29)	.285	1.41 (0.80; 2.51)	.238
Model 2	1.06 (0.80; 1.42)	.683	Ref.	1.13 (0.53; 2.38)	.757	0.57 (0.27; 1.22)	.146	0.68 (0.26; 1.81)	.442	1.74 (0.72; 4.20)	.218
Model 3	1.07 (0.78; 1.45)	.685	Ref.	1.10 (0.52; 2.34)	.808	0.57 (0.26; 1.24)	.158	0.65 (0.22; 1.89)	.427	1.69 (0.69; 4.12)	.249
MI, stroke, and CVD death											
Cases	489		123	128		101		72		65	
Model 1	0.94 (0.81; 1.08)	.376	1 (Ref.)	0.85 (0.59; 1.23)	.390	0.66 (0.45; 0.96)	.030	0.69 (0.45; 1.07)	.100	0.97 (0.62; 1.53)	.908
Model 2	0.93 (0.76; 1.13)	.454	Ref.	0.78 (0.47; 1.30)	.346	0.59 (0.36; 0.98)	0.042	0.60 (0.31; 1.14)	.117	0.94 (0.48; 1.84)	.863
Model 3	0.92 (0.75; 1.13)	.439	Ref.	0.80 (0.48; 1.33)	.390	0.60 (0.36; 1.01)	.053	0.56 (0.28; 1.14)	.108	0.91 (0.47; 1.77)	.777
Stroke and CVD death											
Cases	388		96	104		73		59		56	
Model 1	0.97 (0.83; 1.12)	.652	1 (Ref.)	0.91 (0.62; 1.35)	.647	0.61 (0.41; 0.93)	.021	0.74 (0.46; 1.17)	.197	1.17 (0.73; 1.89)	.517
Model 2	1.01 (0.81; 1.26)	.935	Ref.	0.83 (0.47; 1.46)	.514	0.56 (0.32; 0.99)	.044	0.76 (0.37; 1.55)	.445	1.36 (0.67; 2.78)	.393
Model 3	1.00 (0.79; 1.26	.983	Ref.	0.84 (0.47; 1.48)	.539	0.57 (0.32; 1.01)	.055	0.74 (0.34; 1.60)	.442	1.26 (0.62; 2.60)	.524

The *n* does not include overlapping subjects in the subcohort. Model 1 was adjusted for age and stratified by sex. Model 2 was further adjusted for smoking, marital status, physical activity, body mass index, waist-to-height ratio, hypertension, dyslipidaemia, diabetes, and family history of CVD, and stratified by sex, educational level, quartiles of the waist-to-height ratio, and recruitment centre. Model 3 was additionally adjusted for total energy intake, MedDiet adherence (not considering wine), and consumption of grapes and raisins.

CVD, cardiovascular disease; HR, hazard ratio; MI, myocardial infarction.

## Discussion

In this case-cohort study nested within the PREDIMED trial population of older men and women (mean age 68 years) at high cardiovascular risk, we assessed the association of urinary concentrations of tartaric acid, a reliable biomarker of wine consumption, with incident CVD. The novel finding was that participants who at baseline had a urinary concentration of 12–35 µg/mL of tartaric acid exhibited a lower risk of CVD [HR 0.50 (95% CI 0.27; 0.95)], and similar, but weaker, associations were found for participants with an excretion of 3–12 µg/mL [HR 0.62 (95% CI 0.38; 1.00)] as compared to those presenting concentrations < 3 µg/mL or >35 µg/mL (*Structured Graphical Abstract* ). Among individual components of the CVD composite, myocardial infarction showed the main significant inverse association with higher concentrations of urinary tartaric acid. The inverse association between this urinary biomarker of light-to-moderate wine consumption and CVD was also significant in the subgroups of men and participants with diabetes. However, the association between values of this biomarker reflecting light-to-moderate wine consumption and CVD in women was close to the limit of significance. These findings suggest that the bioactive compounds present in wine may play a role in lowering the risk of CVD.

Wine was not the only source of alcohol intake in PREDIMED participants, and exposure to 3–35 glasses of wine per month may reflect a higher total amount of alcohol. Estimating the consumption of alcoholic beverages remains a critical challenge when investigating the health consequences of alcohol, which is currently the subject of strong controversies. Intake of alcohol can be often underestimated when using self-reported FFQ (the conventional method) due to inaccurate recall or even biased perceptions on the social desirability of drinking alcoholic beverages.^7^ Measurement errors can result in incorrect conclusions about the impact of alcohol on health. Vance *et al*.^14^ concluded that underreporting at-risk drinkers could mask the fact that moderate drinking reduces the risk of adverse health outcomes by spuriously inflating the risks of light-to-moderate consumers given that some heavy drinkers will be misclassified as light or moderate drinkers. In the present study, tartaric acid, an objective biomarker, which does not depend on subjective recall or social desirability, was associated with lower rates of CVD risk in the range corresponding to low-to-moderate wine consumption. However, when wine consumption was estimated using self-reported questionnaires, we did not observe any significant inverse association within this range. Therefore, the use of a more objective biological marker for wine consumption, in conjunction with the self-reported data obtained using FFQ, could represent a significant advancement in studying the impact of alcohol on health. In our study, tartaric acid excreted in urine predicted wine consumption. This is consistent with data from previous studies reporting a direct linear association between urinary tartaric acid and wine consumption.^9,10^ As expected, we observed a significant variation in urine tartaric acid concentrations among participants related to their consumption of wine.

The observed association between wine consumption and a lower incidence of cardiovascular events in the group with moderate levels of tartaric acid in their urine should be solely attributed to their wine consumption. Although the consumption of grapes, raisins, and grape juice can increase tartaric acid concentrations in urine, the intake of these foods among PREDIMED participants was very low, and differences among groups were nutritionally negligible. Additionally, the consumption of grape juice was almost null in our participants. Previous studies have demonstrated a clear dose–response relationship between urinary tartaric acid and wine consumption, indicating that it is a robust marker of actual intake.^9^ Given the stability of urinary tartaric acid concentrations across individuals demonstrated in previous studies, the specificity of its dietary origin, the high sensitivity of the analytical method (with only 2 samples falling below the limit of quantification), and the scarcity in the consumption of its alternative dietary sources, tartaric acid can be considered as a reliable and objective biomarker of wine consumption.^15^

To our knowledge, this is the first case-cohort study of CVD assessing and quantifying the effects of wine consumption measured with an objective biomarker. In our study, light-to-moderate consumption of wine was associated with a lower risk of CVD. Prior epidemiologic studies have also reported a beneficial relationship of wine consumption with cardiovascular health, but with difficulties and potential problems in the quantification of intake.^6^ A study carried out in a Norwegian cohort of more than 115 000 men and women followed up for an average of 16 years showed an inverse association between wine consumption and CVD mortality.^16^ In a cohort that included participants free of CVD, moderate wine consumption was associated with better cardiovascular health.^17^ Two large observational studies observed a lower risk of coronary heart disease in association with self-reported wine intake.^18,19^ An intervention study in healthy adults reported an improvement in cardiovascular risk factors, fasting high-density lipoprotein cholesterol (HDL-c), and fibrinogen after four weeks of consuming 200–300 mL/day of red wine.^20^ However, the doses at which wine might benefit cardiovascular health are controversial. A meta-analysis reported a J-shaped relationship between wine consumption and cardiovascular mortality,^21^ emphasizing the salutary effects of light-to-moderate consumption, but not of heavy drinking. Our results support this dose–response pattern, as we observed that high levels of urinary tartaric acid were not associated with lower CVD risk. However, moderate consumption has been defined as 1–2 drinks/day,^22^ which aligns with the higher doses we identified as associated with a reduced risk of CVD. However, we also observed a lower risk of CVD at lower doses. It is important to consider that our analysis focused exclusively on measuring wine consumption, without adding ethanol from other alcoholic beverages, which may explain the low doses at which the benefits were observed. Nevertheless, previous literature has not reached a consensus on which doses have the most favourable effect on cardiovascular health. Jespersen *et al*.^23^ reported that <1 glass per day was associated with the lowest odds of CVD in patients with chronic kidney disease. A large prospective study that included more than 380 000 men and women in Europe found a U-shaped association between alcohol (or wine consumption) and all-cause mortality. Interestingly, lifetime never alcohol users presented a higher risk of death compared to moderate drinkers (0.1–2.9 g/day).^24^ On the other hand, Mukamal *et al*.^18^ reported that consumption of two or more drinks per day (considering wine, beer, and liquor) was associated with lower risk of coronary heart disease in an older population. Another observational study that included older men reported reduced all-cause and CVD mortality with long-term light consumption of wine, equivalent to less than half a glass per day,^25^ which agrees with our findings. Furthermore, that study emphasized the effects of long-term consumption of alcohol on cardiovascular health, which also concurs with our results and previous independent findings.^26^ In addition, as the results for changes included in the Supplemental material show, our results indicated that usual consumption of wine in real-life conditions (baseline values, reflecting customary intake) may have a more significant beneficial impact on CVD rates than short-term changes.

On the other hand, Mendelian randomization studies have generally found a null or positive linear association between alcohol consumption and CVD rates.^27,28^ These studies offer the advantage of minimizing confounding variables and ruling out potential reverse causation. However, they cannot differentiate between types of alcohol, and they also lack the ability to evaluate drinking patterns.^29^ In addition, Mendelian randomization studies are not able to consider the interactions between diet, foods, and nutrients on the incidence of chronic diseases (i.e. epigenetic effects of Mediterranean diet on incidence of CVD events).^30^ Therefore, these studies may overlook or decry the beneficial effects of bioactive compounds found in wine compared to other distilled alcoholic beverages, depending on the dietary pattern in which they are consumed.

Regarding the differences of wine consumption between men and women, conflicting results have been reported in the literature. A meta-analysis that included 84 studies over the last 30 years found no differences between men and women, and reported a protective effect of alcohol against CVD for both sexes.^31^ This is in line with our results, since we did not find significant differences between sexes in the association of tartaric acid with CVD.

The mechanisms underlying the potential beneficial effects of wine remain uncertain, whether they are attributed to its ethanol content or other nutritional components, such as polyphenols. Most studies support the hypothesis that wine and its bioactive compounds confer benefits independently of ethanol, as wine has exhibited a superior effect compared to other alcoholic beverages.^16,32,33^ The main group of bioactive compounds present in wine is polyphenols, especially malvidin, procyanidin, catechin, and tyrosol.^34,35^ Polyphenol intake was associated with a reduced incidence of CVD events in the PREDIMED trial cohort.^36^ Polyphenols have been demonstrated to exert a variety of health benefits that could confer cardioprotective properties to wine, such as anti-inflammatory properties.^37–39^ Therefore, multiple mechanisms may explain the cardioprotective properties of polyphenols.

Our study has several strengths. First, wine consumption was estimated based on the concentrations of tartaric acid in urine, providing an objective and reliable measure of actual consumption. Second, we designed a case-cohort study in a well-known large long-term intervention trial that enabled us to explore the effects of wine consumption on CVD rates while accounting for potential confounding factors, and to test the interaction by intervention group. There are also limitations to our study. Since participants were older individuals at high risk of CVD living in a Mediterranean country, results may not be generalized to other populations. Even though we adjusted for several potential confounders, residual confounding cannot be discounted. While the observational design of our study limits our ability to establish causality, the use of a prospective case-cohort design with long follow-up reduced the likelihood of reverse causation. Finally, the determination of tartaric acid only indirectly measured wine consumption, thereby excluding other alcoholic beverages from the analysis.

In conclusion, using an objective and reliable urinary biomarker, we found that light-to-moderate consumption of wine was associated with a lower rate of clinical cardiovascular events in a Mediterranean population at high cardiovascular risk.

## Supplementary Material

ehae804_Supplementary_Data

## Declarations

### Data Availability

The data that support the findings of this study are available from the corresponding author, upon reasonable request.

### Pre-registered Clinical Trial Number

This trial was registered in Current Controlled Trials, London (ISRCTN35739639, funded by Instituto de Salud Carlos III, Spanish Government).
