# Supplementary material for: Urinary tartaric acid as a biomarker of wine consumption and cardiovascular risk: the PREDIMED trial
Source: Eur Heart J. 2024 Dec 18;46(2):161–72. doi: 10.1093/eurheartj/ehae804 (PMC11704392; doi:10.1093/eurheartj/ehae804)
Supplement: ehae804_Supplementary_Data [file ehae804_supplementary_data.pdf]

Urinary tartaric acid as a biomarker of wine consumption and cardiovascular risk: the PREDIMED trial

Inés Domínguez-López[1,2,3†], Rosa M Lamuela-Raventós[1,2,3†], Cristina Razquin[3,4], Camila Arancibia-Riveros[1,2], Polina Galkina[1,2,3], Jordi Salas-Salvadó[3,5], Ángel M Alonso-Gómez[3,6], Montserrat Fitó[3,7], Miquel Fiol[3,8], José Lapetra[3,9], Enrique Gómez-Gracia[10], José V Sorlí[,11], Miguel Ruiz-Canela[3,4], Olga Castañer[3,7], Liming Liang[12,13], Lluis Serra-Majem[14], Frank B Hu[15], Emilio Ros[3,16], Miguel Ángel Martínez-González[3,4*], Ramon Estruch[3,17*]

Expanded Methods

1. *Study design*

The trial was conducted in Spain from October 2003 to December 2010 and involved 7,447 participants at high cardiovascular risk (www.predimed.es). This trial was registered in Current Controlled Trials, London (ISRCTN35739639, funded by Instituto de Salud Carlos III, Spanish Government).

Eligible participants included men (aged 55-80 years) and women (aged 60-80 years) with type-2 diabetes or exhibiting at least three of the following risk factors: current smoking, hypertension, dyslipidaemia, overweight/obesity, and/or a family history of premature CVD. Detailed methodology and participant criteria have been previously published [1,2].

Food consumption was estimated using a validated, semi-quantitative food frequency questionnaire comprising 137 items, including wine consumption, with the guidance of skilled dietitians [3]. To determine nutrient intakes, Spanish food composition tables were utilized. To evaluate compliance with the MedDiet, a 14-questionnaire was employed, assigning a score of either 0 or 1 for each dietary component [1]. To address the potential confounding effects related to the overall dietary pattern, we incorporated this score as a

covariate in multivariable models. Consequently, in this study, we excluded the question pertaining to wine consumption, focusing solely on a 13-point score, ranging from 0 to 13 (minimum score = 0, maximum score = 13). For participants in the MedDiet intervention groups, only habitual drinkers were advised to consume moderate amounts of wine with meals (1 glass/d), while non-drinkers did not receive any recommendation. High-alcohol drinkers were advised to reduce their alcohol intake to moderate consumption levels.

Anthropometric measurements, including weight and height, were taken by trained staff using established techniques, enabling the calculation of body mass index (BMI) in kg/m². To assess physical activity levels, a validated Spanish version of the Minnesota physical activity questionnaire was used, measuring metabolic equivalent tasks per minutes per day (METs min/day) [4].

2. *Urinary tartaric acid measurement*

a. *Reagents and standards*

Formic acid (approximately 98 %) was purchased from Panreac (Barcelona, Spain). L-(+)-Tartaric acid was obtained from Sigma (St Louis, USA). The labelled internal standard DL-(+)-tartaric-2,3-d2 acid was purchased from C/D/N Isotopes (Quebec, Canada). Solvents were high-performance liquid chromatography grade, and all other chemicals were analytical reagent grade. Ultrapure water was obtained from a Milli-Q Gradient water purification system (Millipore, Burlington, USA).

b. *Sample preparation*

Biological samples were collected after an overnight fast, coded, and stored at −80 °C until analysis. Tartaric acid in urine was determined following a validated stable-isotope dilution LC-ESI-MS/MS method with minor modifications[5]. A total of 20 µL of urine

were diluted 1:50 (vol:vol) with 0.5% formic acid in water, and 10 µL of the internal standard DL-(±)-tartaric-2,3-d2 acid were added. The diluted sample was filtered by 0.20 µm and analyzed by LC–ESI-MS/MS.

c. LC–ESI-MS/MS analysis

The analysis was performed using an Atlantis TE C18, 100 x 2.1 mm, 3 µm (Waters, Milford, MA, USA) reversed-phase column coupled for detection to a triple quadrupole mass spectrometer API 4000 (Applied Biosystems, Foster City, CA, USA). The mass spectrometer was operated in negative electrospray ionisation. The column was maintained at 25 ºC throughout the analysis. Mobile phases A and B were, 0.5% formic acid in water and 0.5% formic acid in acetonitrile, respectively. The following linear gradient was used: held at 100%A for 3.7 min, decreased to 10%A and held for 1.5 min, then returned to initial conditions for 1.8 min. The flow rate was set at 350 µL/min and the injection volume was 2 µL. Post-column addition of acetonitrile (250 µL/min) was carried out to improve analyte ionization efficiency. The detection was accomplished in the multiple reaction monitoring (MRM) mode, and the following MS/MS transitions were used for quantification and confirmation, respectively: m/z 149/87 and m/z 149/73 for tartaric acid, and m/z 151/88 and m/z 151/74 for the tartaric deuterated isotope. The coefficient of variation of the quality controls used in the analysis was 3.25%. Tartaric acid was quantified using the MultiQuant Software. Two samples that were under the limit of quantification were replaced by the midpoint between the limit of detection and the limit of quantitation.

References

1. Martínez-González MÁ, Corella D, Salas-salvadó J, et al. Cohort profile: Design and methods of the PREDIMED study. *Int J Epidemiol*. 2012;41:377–385.

2. Estruch R, Ros E, Salas-Salvadó J, et al. Primary Prevention of Cardiovascular Disease with a Mediterranean Diet Supplemented with Extra-Virgin Olive Oil or Nuts. *New England Journal of Medicine*. 2018;378:e34.

3. Fernández-Ballart JD, Piñol JL, Zazpe I, et al. Relative validity of a semi-quantitative food-frequency questionnaire in an elderly Mediterranean population of Spain. *British Journal of Nutrition*. 2010;103:1808–1816.

4. Elosua R, Garcia M, Aguilar A, Molina L, Covas MI, Marrugat J. Validation of the Minnesota leisure time physical activity questionnaire in Spanish women. *Med Sci Sports Exerc*. 2000;32:1431–1437.

5. Regueiro J, Vallverdú-Queralt A, Simal-Gándara J, Estruch R, Lamuela-Raventós R. Development of a LC-ESI-MS/MS approach for the rapid quantification of main wine organic acids in human urine. *J Agric Food Chem*. 2013;61:6763–6768.

SUPPLEMENTARY FIGURES

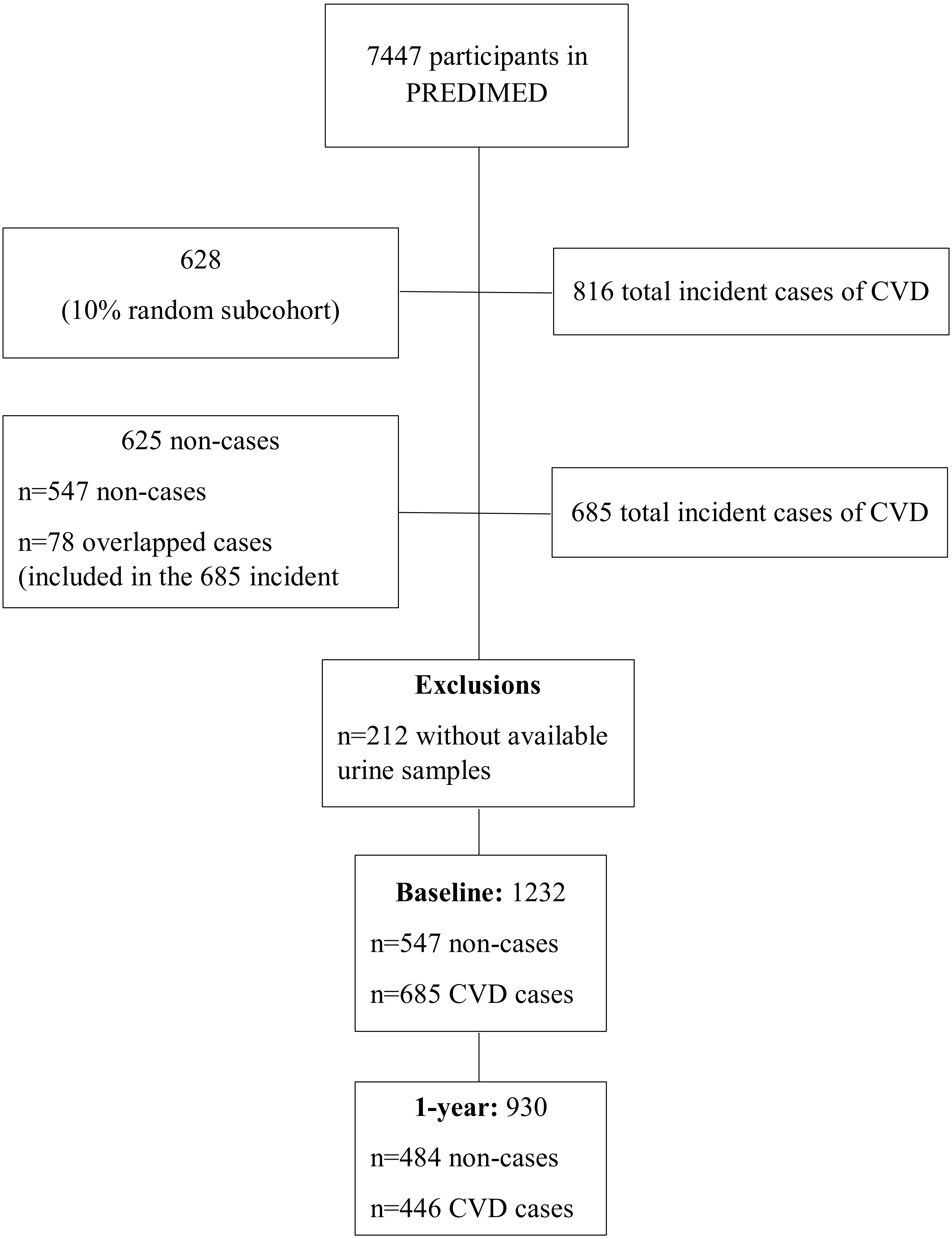

Figure S1. Flowchart of study participants.

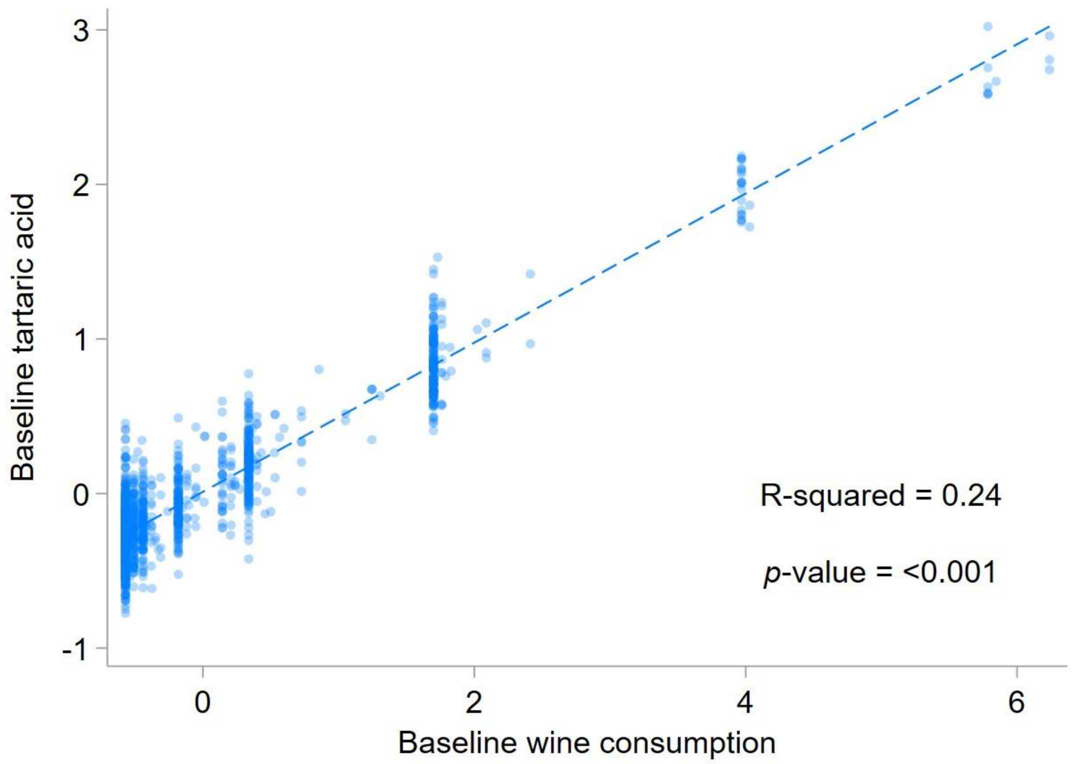

Figure S2. Multivariate-adjusted regression between baseline wine consumption and urinary tartaric acid. An inverse normal transformation was applied to urinary tartaric acid, whereas wine consumption was z-scaled. Models were adjusted for age, sex, educational level, smoking, physical activity, BMI, diabetes, dyslipidaemia, hypertension, total energy intake, MedDiet adherence (not considering wine), and consumption of grapes and raisins.

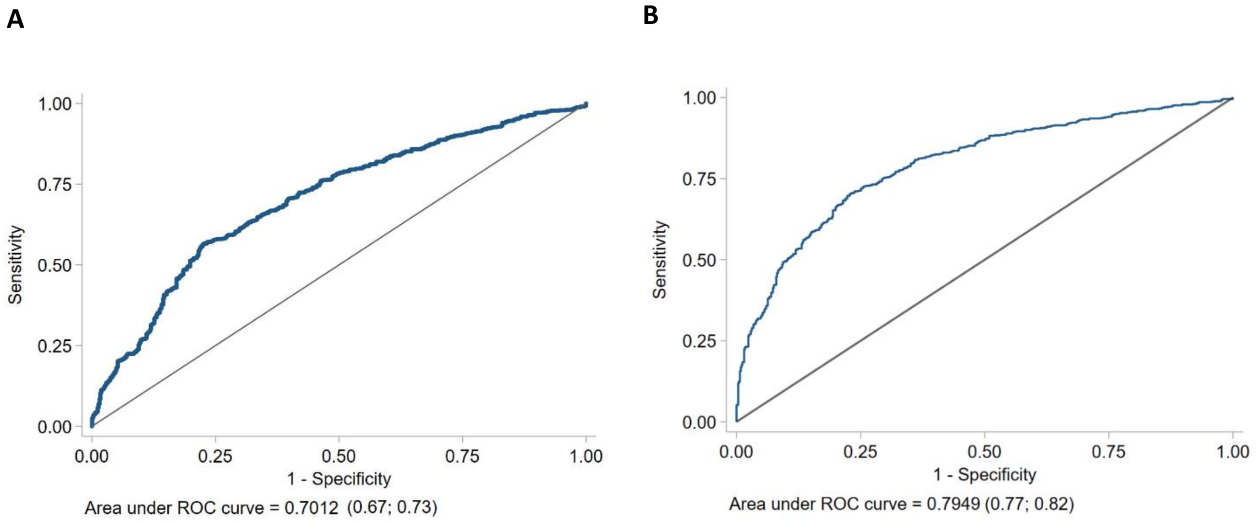

Figure S3. Receiver operating characteristic (ROC) curves for prediction of baseline wine consumption (yes/no) by baseline urinary tartaric acid without adjusting (A) and adjusted for potential confounders (B).

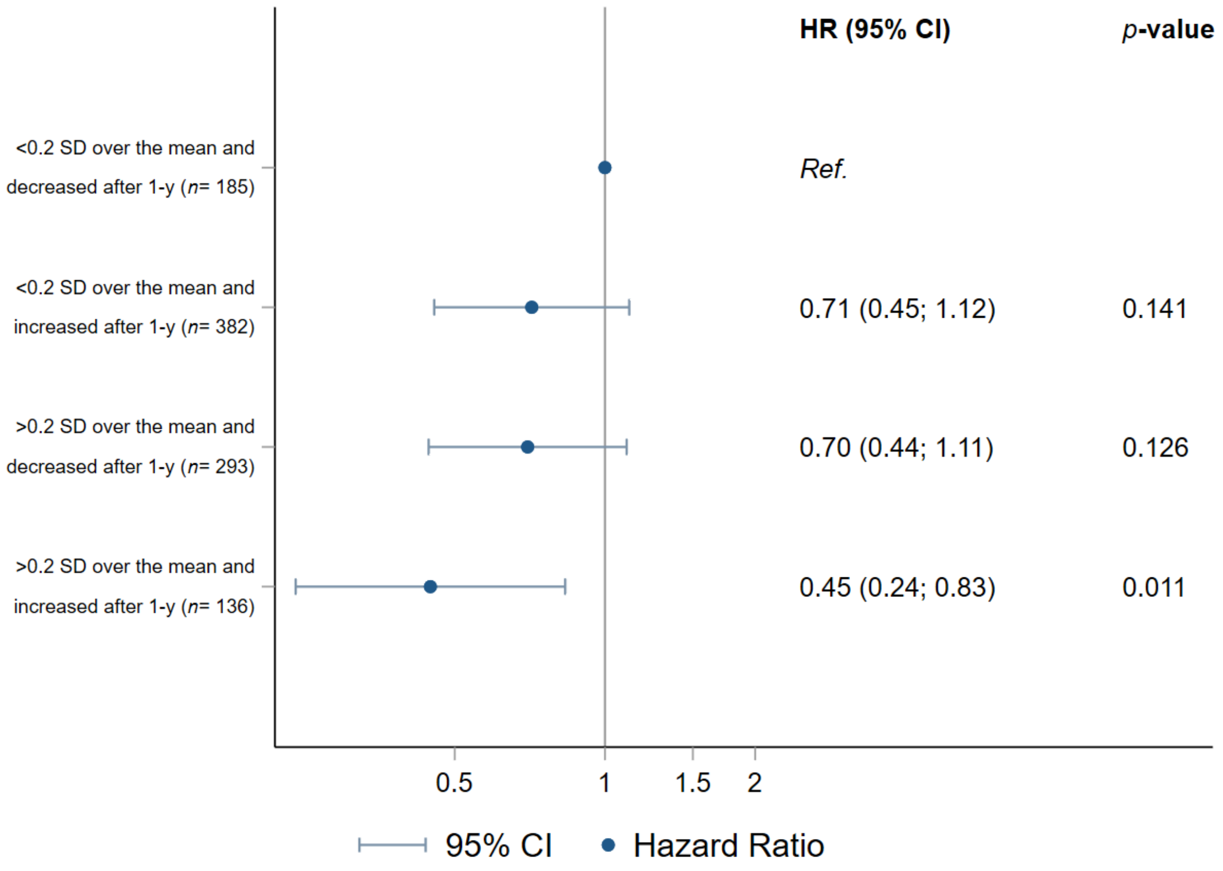

Figure S4. Multivariable adjusted HRs (95% CI) of CVD by the joint classification according to baseline levels and 1-year changes in urinary tartaric acid. HR, hazard ratio; CVD, cardiovascular disease.

SUPPLEMENTARY TABLES

Table S1. Dietary intake at baseline according to categories of tartaric acid excretion.

	<1 µg/mL (*n* = 288)	1 - 3 µg/mL (*n* = 336)	3 - 12 µg/mL (*n* = 280)	12 - 35 µg/mL (*n* = 164)	>35 µg/mL (*n* = 153)	*p*-value
Carbohydrate (g/d)	236.3 ± 75.6	241.6 ± 81.9	239.2 ± 77.7	236.1 ± 80.6	247.8 ± 89.9	0.629
Protein (g/d)	91.5 ± 22.9	94.6 ± 23.9	95.4 ± 24.5	93.3 ± 22.6	94.3 ± 25.3	0.356
Total fat (g/d)	97.2 ± 30.3	100.5 ± 32.3	101.6 ± 33.1	102.3 ± 27.8	100.4 ± 29.2	0.392
Saturated fat (g/d)	25.1 ± 8.7	26.1 ± 10.0	26.7 ± 9.9	26.2 ± 8.8	26.5 ± 8.8	0.304
Monounsaturated fat (g/d)	48.3 ± 16.3	49.5 ± 16.5	50.6 ± 17.9	51.3 ± 14.6	49.3 ± 14.4	0.317
Polyunsaturated fat (g/d)	15.1 ± 6.4	15.9 ± 7.3	16.0 ± 7.2	16.0 ± 7.0	16.4 ± 7.3	0.378
Vegetables (g/d)	336.7 ± 168.6	345.2 ± 171.7	331.1 ± 133.1	334.8 ± 134.0	326.4 ± 155.6	0.728
Fruits (g/d)	381.9 ± 223.6	383.3 ± 203.9	372.4 ± 194.0	381.8 ± 226.5	384.8 ± 240.5	0.969
Legumes (g/d)	20.0 ± 20.6	19.6 ± 12.3	20.1 ± 12.1	19.6 ± 9.1	20.3 ± 11.5	0.986
Nuts (g/d)	8.9 ± 12.3	10.2 ± 14.1	10.2 ± 13.4	10.8 ± 16.5	12.1 ± 12.5	0.210
Fish and seafood (g/d)	95.8 ± 45.5	105.1 ± 52.1	106.6 ± 76.1	102.0 ± 50.2	100.9 ± 46.9	0.171
Meat/meat products (g/d)	130.1 ± 66.1	134.3 ± 58.3	141.9 ± 61.5	138.8 ± 55.3	149.0 ± 61.3	0.016
Dairy products (g/d)	415.1 ± 236.2	406.1 ± 223.3	388.9 ± 220.1	343.2 ± 200.0	302.9 ± 198.0	<0.001
Cereals (g/d)	143.7 ± 82.7	145.9 ± 87.7	141.0 ± 81.7	147.5 ± 89.5	151.7 ± 88.8	0.778
Pastries (g/d)	20.5 ± 24.6	24.2 ± 30.0	22.2 ± 30.4	19.1 ± 24.2	23.2 ± 28.3	0.301
Virgin olive oil (g/d)	21.9 ± 23.9	20.9 ± 23.5	23.7 ± 24.5	27.4 ± 24.6	21.3 ± 21.2	0.045

Values are means ± standard deviation (SD).
Statistical analyses were undertaken using one-ANOVA factor.
$p < 0.05$ was considered significant.

Table S2. Multivariable-adjusted linear regression assessing the association between baseline wine consumption (mL/d, independent variable) and urinary concentrations of tartaric acid (μg/mL, outcome), *n* = 1232.

	β (CI 95%) per 1-SD increment	*p*-value	β (CI 95%) for Tertiles of Wine Consumption			
			T1	T2 vs. T1	T3 vs. T1	*p*-trend
Tartaric Acid						
Model 1	0.45 (0.40; 0.51)	<0.001	Ref.	0.23 (-0.10; 0.36)	0.93 (0.80; 1.05)	<0.001
Model 2	0.45 (0.40; 0.50)	<0.001	Ref.	0.22 (0.07; 0.37)	0.92 (0.75; 1.08)	<0.001
Model 3	0.47 (0.41; 0.53)	<0.001	Ref.	0.24 (0.09; 0.39)	0.95 (0.78; 1.12)	<0.001

T, tertile.
Overlapping subjects in the subcohort were excluded from the analysis.
Model 1 was adjusted for age and sex. Model 2 was further adjusted for smoking, educational level, physical activity, BMI, hypertension, dyslipidemia, and diabetes. Model 3 was additionally adjusted for total energy intake, MedDiet adherence (not considering wine), and consumption of grapes and raisins. Robust variance estimators were used to account for recruitment center.
1-SD of wine consumption is equivalent to 103.5 ml/day and 1-SD of urinary tartaric acid is equivalent to 43.7 μg/ml.
$p < 0.05$ were considered significant.

Table S3. Risk of long-term all-cause mortality by categories of baseline urinary tartaric acid concentrations for the total population and stratifying by sex.

	Tartaric acid (µg/mL) per 1-SD		<1 µg/mL (*n* = 291)	1 - 3 µg/mL (*n* = 340)		3 - 12 µg/mL (*n* = 281)		12 - 35 µg/mL (*n* = 164)		>35 µg/mL (*n* = 156)	
	HR (CI 95%)	*p*-value		HR (CI 95%)	*p*-value	HR (CI 95%)	*p*-value	HR (CI 95%)	*p*-value	HR (CI 95%)	*p*-value
Total											
Cases	534		132	143		105		81		73	
Person-years	15517		3662	4312		3617		1994		1932	
Model 1	0.99 (0.90; 1.08)	0.770	1 (Ref.)	1.00 (0.80; 1.27)	0.970	0.79 (0.61; 1.03)	0.079	0.98 (0.74; 1.31)	0.915	1.12 (0.84; 1.48)	0.441
Model 2	0.99 (0.90; 1.09)	0.835	Ref.	1.03 (0.81; 1.30)	0.823	0.82 (0.64; 1.06)	0.127	1.06 (0.77; 1.45)	0.729	1.04 (0.76; 1.43)	0.814
Model 3	0.98 (0.89; 1.08)	0.670	Ref.	1.03 (0.81; 1.30)	0.822	0.82 (0.63; 1.06)	0.129	1.03 (0.75; 1.42)	0.846	0.99 (0.71; 1.38)	0.956
Men											
Cases	282		54	67		54		57		50	
Person-years	7010		1278	1637		1646		1204		1244	
Model 3	0.99 (0.88; 1.14)	0.976	Ref.	1.16 (0.80; 1.67)	0.430	0.81 (0.55; 1.18)	0.271	1.16 (0.76; 1.76)	0.490	0.92 (0.58; 1.44)	0.718
Women											
Cases	252		78	76		51		24		23	
Person-years	8508		2384	2675		1971		790		688	
Model 3	0.95 (0.81; 1.11)	0.506	Ref.	0.96 (0.69; 1.32)	0.792	0.83 (0.58; 1.18)	0.294	0.87 (0.51; 1.50)	0.627	1.11 (0.64; 1.94)	0.701

The follow-up for all-cause mortality continued until December 31, 2020, with a mean duration of 12.6 years.
Overlapping subjects in the subcohort were excluded from the analysis.
HR, hazard ratio.
Model 1 was adjusted for age and stratified by sex. Model 2 was further adjusted for smoking, marital status, physical activity, educational level, BMI, waist-to-height ratio, hypertension, dyslipidemia, diabetes, and family history of CVD, and stratified by sex, quartiles of the waist-to-height ratio and recruitment centre. Model 3 was additionally adjusted for total energy intake, MedDiet adherence (not considering wine), and consumption of grapes and raisins. The *p* value for interaction (sex * category of the joint combination of baseline levels of tartaric acid, with 4 degrees of freedom) was derived from a Cox model adjusted as model 3, and it was not statistically significant $p_{\text{for interaction}} = 0.53$.

Table S4. Risk of CVD by categories of baseline wine consumption for the total population.

	Wine consumption (mL/day)		<1 glasses of wine/mo (*n* = 561)	1 - 3 glasses of wine/mo (*n* = 174)		3 - 12 glasses of wine/mo (*n* = 136)		12 - 35 glasses of wine/mo (*n* = 201)		>35 glasses of wine/mo (*n* = 160)	
	HR (CI 95%)	*p*-value		HR (CI 95%)	*p*-value	HR (CI 95%)	*p*-value	HR (CI 95%)	*p*-value	HR (CI 95%)	*p*-value
Total											
Cases	685		315	95		67		115		93	
Person-years	6412		2871	867		788		1043		843	
Model 1	0.94 (0.82; 1.07)	0.346	1 (Ref.)	1.17 (0.81; 1.70)	0.405	0.92 (0.62; 1.36)	0.661	0.87 (0.62; 1.23)	0.438	0.87 (0.60; 1.25)	0.447
Model 2	0.88 (0.71; 1.08)	0.205	Ref.	1.91 (1.11; 3.27)	0.019	1.01 (0.56; 1.84)	0.968	1.33 (0.85; 2.08)	0.215	0.71 (0.40; 1.24)	0.227
Model 3	0.87 (0.71; 1.08)	0.222	Ref.	1.85 (1.07; 3.19)	0.027	1.02 (0.57; 1.85)	0.943	1.53 (0.96; 2.45)	0.075	0.67 (0.37; 1.23)	0.199

HR, hazard ratio.
The *n* does not include overlapping subjects in the subcohort.
Model 1 was adjusted for age and stratified by sex. Model 2 was further adjusted for smoking, marital status, physical activity, educational level, BMI, waist-to-height ratio, hypertension, dyslipidemia, diabetes, and family history of CVD, and stratified by sex, quartiles of the waist-to-height ratio and recruitment centre. Model 3 was additionally adjusted for total energy intake, MedDiet adherence (not considering wine), and consumption of grapes and raisins.

Table S5. Risk of CVD by categories of baseline urinary tartaric acid concentrations stratifying by diabetes.

	<1 µg/mL (*n* = 291)	1 - 3 µg/mL (*n* = 340)		3 - 12 µg/mL (*n* = 281)		12 - 35 µg/mL (*n* = 164)		>35 µg/mL (*n* = 156)	
		HR (CI 95%)	*p*-value	HR (CI 95%)	*p*-value	HR (CI 95%)	*p*-value	HR (CI 95%)	*p*-value
No diabetes									
Cases	55	64		58		43		44	
Model 1	1 (Ref.)	0.86 (0.50; 1.46)	0.566	0.72 (0.42; 1.25)	0.242	0.96 (0.53; 1.73)	0.880	1.34 (0.72; 2.51)	0.359
Model 2	Ref.	1.55 (0.62; 3.88)	0.350	1.10 (0.37; 3.26)	0.857	1.54 (0.49; 4.87)	0.463	2.87 (0.96; 8.59)	0.060
Model 3	Ref.	1.53 (0.56; 4.19)	0.413	1.13 (0.36; 3.48)	0.836	1.36 (0.39; 4.74)	0.633	3.01 (0.93; 9.71)	0.065
Diabetes									
Cases	114	124		85		51		47	
Model 1	1 (Ref.)	1.07 (0.68; 1.68)	0.767	0.72 (0.46; 1.15)	0.171	0.57 (0.33; 1.00)	0.051	0.98 (0.54; 1.76)	0.938
Model 2	Ref.	0.72 (0.34; 1.50)	0.378	0.44 (0.21; 0.89)	0.022	0.48 (0.17; 1.36)	0.166	0.61 (0.23; 1.65)	0.334
Model 3	Ref.	0.88 (0.40; 1.96)	0.752	0.45 (0.21; 0.95)	0.036	0.47 (0.15; 1.48	0.200	0.60 (0.22; 1.63)	0.315

HR, hazard ratio.

The *n* does not include overlapping subjects in the subcohort.

Model 1 was adjusted for age and stratified by sex. Model 2 was further adjusted for smoking, marital status, physical activity, BMI, waist-to-height ratio, hypertension, dyslipidemia, and family history of CVD, and stratified by sex, educational level, quartiles of the waist-to-height ratio and recruitment center. Model 3 was additionally adjusted for total energy intake, MedDiet adherence (not considering wine), and consumption of grapes and raisins.

The *p* value for interaction (diabetes * category of the joint combination of baseline levels of tartaric acid, with 4 degrees of freedom) was derived from a Cox model adjusted as model 3, and it was not statistically significant $p_{\text{for interaction}}$ =0.056.

Table S6. General characteristics of the study population at baseline according to categories of changes in tartaric acid (*n* = 930).

	<3 µg/mL of baseline tartaric that decreased after 1-y (*n* = 140)	<3 µg/mL of baseline tartaric acid that increased after 1-y (*n* = 329)	>3 µg/mL of baseline tartaric acid that decreased after 1-y (*n* = 303)	>3 µg/mL of baseline tartaric acid that increased after 1-y (*n* = 158)	*p*-value
Δ Urinary tartaric acid, µg/mL	-0.5 (-1.0- -0.2)	2.4 (0.6-8.0)	-10.3 (-29.2- -3.6)	15.6 (6.7-48.9)	-
Age, years	68.6 ± 6.0	68.1 ± 6.2	67.9 ± 5.9	68.3 ± 6.7	0.721
Women, n (%)	93 (65.5)	198 (59.5)	139 (45.3)	62 (39.2)	<0.001
BMI, kg/m^2	30.4 ± 3.8	29.9 ± 3.8	29.9 ± 3.4	29.5 ± 3.3	0.231
Diabetes Mellitus, n (%)	79 (55.6)	179 (53.8)	159 (51.8)	68 (43.0)	0.103
Dyslipidemia, n (%)	94 (66.2)	241 (72.4)	197 (64.2)	112 (70.9)	0.124
Hypertension, n (%)	127 (89.4)	273 (82.0)	253 (82.4)	129 (81.7)	0.194
Educational level, n (%)					0.364
Low	115 (81.0)	258 (77.5)	241 (78.5)	115 (72.8)	
High & medium	27 (19.0)	75 (22.5)	66 (21.5)	43 (27.2)	
Smoking habit, n (%)					0.002
Current smokers	12 (8.5)	38 (11.4)	46 (15.0)	31 (19.6)	
Former smokers	31 (21.8)	86 (25.8)	99 (32.3)	46 (29.1)	
Total energy intake, kcal/day	2205 ± 592	2266 ± 580	2328 ± 613	2422 ± 677	0.010
Physical activity, METS·min/day	223 ± 214	224 ± 205	256 ± 243	301 ± 263	0.003
Family history of early-onset CHD, n (%)	28 (19.7)	79 (23.7)	69 (22.5)	36 (22.8)	0.820
Intervention group, n (%)					0.360
MedDiet + EVOO	51 (35.9)	117 (35.1)	106 (34.5)	65 (41.1)	
MedDiet + nuts	39 (27.5)	110 (33.0)	112 (36.5)	50 (31.7)	
Control diet	52 (36.6)	106 (31.8)	89 (29.0)	43 (27.2)	
Wine consumption, mL/day	17 ± 34	25 ± 50	95 ± 126	125 ± 143	<0.001
Grapes & raisins (g/d)	11 ± 19	13 ± 24	16 ± 28	19 ± 37	0.033

BMI, body mass index; METS, metabolic task equivalents; CVD, cardiovascular disease; MedDiet, Mediterranean diet; EVOO, extra-virgin olive oil. Values are percentages for categorical variables and means ± SD for continuous variables. Tartaric acid concentrations are presented as median values (Q1-Q3).
The *n* does not include overlapping subjects in the subcohort.
One-ANOVA factor was used for continuous variables, and a chi-square test was used for categorical variables.
$p < 0.05$ was considered significant.

Table S7. Multivariable adjusted linear regression assessing the association between 1-year changes in wine consumption (wine changes=independent variable, mL/d) and 1-y changes in urinary concentrations of tartaric acid (outcome, µg/mL), n = 930.

	β (CI 95%) per 1-SD increment	p-value	β (CI 95%) for 1-y Changes in Wine Consumption (Tertiles)			
			T1	T2 vs. T1	T3 vs. T1	p-trend
Outcome: 1-y Changes in Tartaric Acid						
Model 1	5.58 (2.31; 8.86)	0.001	Ref.	4.13 (-4.57; 12.82)	9.00 (0.57; 17.44)	0.037
Model 2	5.30 (-2.96; 13.56)	0.177	Ref.	3.57 (-15.85; 23.00)	7.98 (-11.43; 27.40)	0.371
Model 3	5.26 (-3.10; 13.62)	0.185	Ref.	3.22 (-15.17; 21.62)	7.22 (-11.84; 26.27)	0.408

T, tertile.
Overlapping subjects in the subcohort were excluded from the analysis.
One-year changes in wine consumption and urinary tartaric acid were analyzed using the same models as for the baseline values but they were further adjusted for baseline values of tartaric acid and wine consumption, and for the intervention group in the trial (MedDiet+EVOO; MediDiet+nuts; control diet). Model 1 was adjusted for age, sex, and baseline values. Model 2 was further adjusted for smoking, educational level, physical activity, BMI, hypertension, dyslipidemia, diabetes, and intervention group. Model 3 was additionally adjusted for total energy intake, MedDiet adherence (not considering the score given by wine), and consumption of grapes and raisins. Robust variance estimators were used to account for recruitment center.

1-SD of 1-y changes in wine consumption is equivalent to 83.1 ml/day.

Table S8. Risk of CVD by 1-y changes in urinary tartaric acid after one year of follow-up for the total population and stratifying by sex.

	1-year changes in tartaric acid (µg/mL) per 1-SD<br><br>HR (CI 95%)	<3 µg/mL of baseline tartaric and decreased after 1-y (*n* = 140)	<3 µg/mL of baseline tartaric acid that increased after 1-y (*n* = 329)<br><br>HR (CI 95%)	>3 µg/mL of baseline tartaric acid that decreased after 1-y (*n* = 303)<br><br>HR (CI 95%)	>3 µg/mL of baseline tartaric acid that increased after 1-y (*n* = 158)<br><br>HR (CI 95%)
Total					
Cases	446	72	154	150	70
Model 1	0.94 (0.82; 1.07)	1 (Ref.)	0.78 (0.51; 1.19)	0.75 (0.49; 1.14)	0.58 (0.35; 0.94)
Model 2	0.91 (0.78; 1.07)	Ref.	0.68 (0.42; 1.11)	0.63 (0.39; 1.01)	0.44 (0.24; 0.80)
Model 3	0.89 (0.76; 1.05)	Ref.	0.63 (0.38; 1.03)	0.60 (0.37; 0.98)	0.41 (0.22; 0.76)
Men					
Cases	243	28	74	91	50
Model 3	0.95 (0.75; 1.20)	Ref.	0.91 (0.37; 2.27)	0.78 (0.33; 1.84)	0.54 (0.20; 1.49)
Women					
Cases	203	44	80	59	20
Model 3	0.84 (0.63; 1.11)	Ref.	0.37 (0.19; 0.71)	0.32 (0.16; 0.65)	0.20 (0.07; 0.56)

HR, hazard ratio.

The *n* does not include overlapping subjects in the subcohort.

Model 1 was adjusted for age and stratified by sex. Model 2 was further adjusted for smoking, educational level, marital status, physical activity, BMI, waist-to-height ratio, hypertension, dyslipidaemia, diabetes, family history of CVD and randomized intervention group, and it was stratified by sex and recruitment centre. Model 3 was additionally adjusted for total energy intake, MedDiet adherence (not considering wine), and consumption of grapes and raisins. The p value for interaction (sex * category of the joint combination of baseline levels and changes of tartaric acid, with 3 degrees of freedom) was derived from a Cox model adjusted as model 3, and it was not statistically significant $p_{\text{for interaction}}$ =0.40.

Table S9. Risk of CVD by changes in urinary tartaric acid after one year of follow-up for the total population and stratifying by diabetes.

	<3 µg/mL of baseline tartaric that decreased after 1-y (*n* = 140)	<3 µg/mL of baseline tartaric acid that increased after 1-y (*n* = 329)		>3 µg/mL of baseline tartaric acid that decreased after 1-y (*n* = 303)		>3 µg/mL of baseline tartaric acid that increased after 1-y (*n* = 158)	
		HR (CI 95%)	*p*-value	HR (CI 95%)	*p*-value	HR (CI 95%)	*p*-value
No diabetes							
Cases	28	59		63		35	
Model 1	1 (Ref.)	0.85 (0.45; 1.61)	0.616	0.95 (0.51; 1.78)	0.878	0.64 (0.31; 1.29)	0.213
Model 2	Ref.	0.86 (0.35; 2.10)	0.744	1.13 (0.46; 2.78)	0.798	0.54 (0.18; 1.61)	0.273
Model 3	Ref.	0.78 (0.32; 1.88)	0.575	0.98 (0.39; 2.41)	0.957	0.41 (0.13; 1.32)	0.136
Diabetes							
Cases	44	95		87		35	
Model 1	1 (Ref.)	0.74 (0.42; 1.32)	0.311	0.64 (0.36; 1.14)	0.129	0.59 (0.29; 1.21)	0.149
Model 2	Ref.	0.53 (0.23; 1.23)	0.141	0.41 (0.19; 0.90)	0.026	0.45 (0.17; 1.20)	0.111
Model 3	Ref.	0.48 (0.20; 1.15)	0.099	0.35 (0.15; 0.80)	0.013	0.31 (0.10; 0.92)	0.035

HR, hazard ratio.
The *n* does not include overlapping subjects in the subcohort.
Model 1 was adjusted for age and stratified by sex. Model 2 was further adjusted for smoking, educational level, marital status, physical activity, BMI, waist-to-height ratio, hypertension, dyslipidemia, family history of CVD and randomized intervention group, and it was stratified by sex and recruitment center. Model 3 was additionally adjusted for total energy intake, MedDiet adherence (not considering wine), and consumption of grapes and raisins.
The p value for interaction (diabetes * category of the joint combination of baseline levels and changes of tartaric acid, with 3 degrees of freedom) was derived from a Cox model adjusted as model 3, and it was not statistically significant $p_{\text{for interaction}}$ =0.25.

Table S10. Risk of CVD by changes in urinary tartaric acid after one year of follow-up for the total population and stratifying by intervention group.

	<3 µg/mL of baseline tartaric that decreased after 1-y (*n* = 140)	<3 µg/mL of baseline tartaric acid that increased after 1-y (*n* = 329)		>3 µg/mL of baseline tartaric acid that decreased after 1-y (*n* = 303)		>3 µg/mL of baseline tartaric acid that increased after 1-y (*n* = 158)	
		HR (CI 95%)	*p*-value	HR (CI 95%)	*p*-value	HR (CI 95%)	*p*-value
MedDiet groups							
Cases	42	105		109		51	
Model 1	1 (Ref.)	0.85 (0.50; 1.43)	0.540	0.85 (0.51; 1.43)	0.551	0.67 (0.37; 1.21)	0.181
Model 2	Ref.	0.96 (0.50; 1.83)	0.902	0.79 (0.42; 1.46)	0.449	0.72 (0.35; 1.48)	0.365
Model 3	Ref.	0.85 (0.44; 1.65)	0.640	0.69 (0.36; 1.30)	0.248	0.58 (0.27; 1.24)	0.158
Control group							
Cases	30	49		41		19	
Model 1	1 (Ref.)	0.67 (0.32; 1.39)	0.284	0.57 (0.27; 1.22)	0.147	0.44 (0.17; 1.12)	0.086
Model 2	Ref.	0.32 (0.11; 0.93)	0.037	0.43 (0.17; 1.09)	0.076	0.12 (0.02; 0.60)	0.010
Model 3	Ref.	0.32 (0.11; 0.94)	0.039	0.37 (0.14; 0.94)	0.037	0.09 (0.02; 0.47)	0.004

HR, hazard ratio; MedDiet, Mediterranean diet.
The *n* does not include overlapping subjects in the subcohort.
Model 1 was adjusted for age and stratified by sex. Model 2 was further adjusted for smoking, educational level, marital status, physical activity, BMI, waist-to-height ratio, hypertension, dyslipidemia, diabetes, family history of CVD, and it was stratified by sex and recruitment center. Model 3 was additionally adjusted for total energy intake, MedDiet adherence (not considering wine), and consumption of grapes and raisins.
The *p* value for interaction (intervention group * category of the joint combination of baseline levels and changes of tartaric acid, with 3 degrees of freedom) was derived from a Cox model adjusted as model 3, and it was statistically significant $p_{\text{for interaction}} = 0.016$.

Table S11. Risk of cardiovascular events by changes in urinary tartaric acid after one year of follow-up.

	1-year changes in tartaric acid (μg/mL) per 1-SD		<3 μg/mL of baseline tartaric that decreased after 1-y (*n* = 140)	<3 μg/mL of baseline tartaric acid that increased after 1-y (*n* = 329)		>3 μg/mL of baseline tartaric acid that decreased after 1-y (*n* = 303)		>3 μg/mL of baseline tartaric acid that increased after 1-y (*n* = 158)	
	HR (CI 95%)	*p*-value		HR (CI 95%)	*p*-value	HR (CI 95%)	*p*-value	HR (CI 95%)	*p*-value
Heart failure									
Cases	129		28	55		48		21	
Model 1	1.00 (0.81; 1.24)	0.986	1 (Ref.)	0.77 (0.42; 1.38)	0.376	0.64 (0.35; 1.18)	0.152	0.53 (0.26; 1.09)	0.083
Model 2	1.00 (0.77; 1.29)	0.999	Ref.	0.74 (0.34; 1.57)	0.430	0.53 (0.25; 1.15)	0.108	0.41 (0.15; 1.12)	0.083
Model 3	0.99 (0.76; 1.29)	0.950	Ref.	0.69 (0.32; 1.50)	0.349	0.50 (0.23; 1.12)	0.091	0.40 (0.15; 1.10)	0.076
MI									
Cases	71		7	32		24		17	
Model 1	1.11 (0.89; 1.38)	0.345	1 (Ref.)	1.42 (0.58; 3.44)	0.443	1.04 (0.42; 2.59)	0.931	1.35 (0.52; 3.51)	0.539
Model 2	1.12 (0.85; 1.48)	0.414	Ref.	1.20 (0.41; 3.55)	0.740	0.81 (0.26; 2.54)	0.713	1.00 (0.28; 3.59)	0.996
Model 3	1.13 (0.83; 1.54)	0.429	Ref.	1.09 (0.33; 3.33)	0.880	0.81 (0.25; 2.59)	0.716	1.03 (0.28; 3.75)	0.960
Stroke									
Cases	104		20	36		43		13	
Model 1	0.89 (0.70; 1.12)	0.307	1 (Ref.)	0.72 (0.38; 1.36)	0.313	0.80 (0.43; 1.51)	0.499	0.43 (0.19; 0.97)	0.043
Model 2	0.88 (0.66; 1.16)	0.366	Ref.	0.57 (0.25; 1.29)	0.177	0.66 (0.30; 1.44)	0.298	0.27 (0.09; 0.82)	0.020
Model 3	0.80 (0.60; 1.08)	0.145	Ref.	0.45 (0.18; 1.14)	0.094	0.57 (0.24; 1.37)	0.209	0.20 (0.06; 0.68)	0.011
Death from CVD									
Cases	152		28	54		66		30	
Model 1	0.84 (0.69; 1.02)	0.083	1 (Ref.)	0.65 (0.36; 1.21)	0.174	0.74 (0.41; 1.36)	0.338	0.54 (0.27; 1.08)	0.080
Model 2	0.75 (0.59; 0.96)	0.025	Ref.	0.43 (0.22; 0.91)	0.028	0.55 (0.27; 1.11)	0.094	0.38 (0.15; 0.92)	0.033
Model 3	0.77 (0.60; 0.99)	0.042	Ref.	0.43 (0.20; 0.91)	0.027	0.54 (0.26; 1.13)	0.101	0.38 (0.15; 0.97)	0.043
MI+Stroke+CVD death									
Cases	318		54	120		130		57	
Model 1	0.91 (0.79; 1.05)	0.193	1 (Ref.)	0.79 (0.50; 1.25)	0.309	0.80 (0.50; 1.27)	0.350	0.60 (0.35; 1.02)	0.060
Model 2	0.87 (0.73; 1.04)	0.132	Ref.	0.65 (0.37; 1.12)	0.120	0.66 (0.39; 1.11)	0.118	0.43 (0.22; 0.84)	0.013
Model 3	0.85 (0.71; 1.02)	0.084	Ref.	0.59 (0.33; 1.03)	0.065	0.62 (0.36; 1.08)	0.093	0.39 (0.19; 0.78)	0.008
Stroke+CVD death									

Cases	256		48		90		109		43	
Model 1	0.86 (0.73; 1.01)	0.071	1 (Ref.)		0.68 (0.42; 1.12)	0.130	0.77 (0.47; 1.25)	0.291	0.50 (0.28; 0.89)	0.019
Model 2	0.81 (0.66; 0.99)	0.036	Ref.		0.53 (0.29; 0.96)	0.036	0.63 (0.36; 1.10)	0.107	0.35 (0.17; 0.74)	0.006
Model 3	0.79 (0.64; 0.97)	0.021	Ref.		0.47 (0.26; 0.87)	0.017	0.58 (0.32; 1.05)	0.074	0.31 (0.14; 0.68)	0.003

HR, hazard ratio.

The *n* does not include overlapping subjects in the subcohort.

Model 1 was adjusted for age and stratified by sex. Model 2 was further adjusted for smoking, educational level, marital status, physical activity, BMI, waist-to-height ratio, hypertension, dyslipidemia, diabetes, family history of CVD and randomized intervention group, and it was stratified by sex and recruitment center. Model 3 was additionally adjusted for total energy intake, MedDiet adherence (not considering wine), and consumption of grapes and raisins.
